# Mechanotransduction of the vasculature in Hutchinson-Gilford Progeria Syndrome

**DOI:** 10.3389/fphys.2024.1464678

**Published:** 2024-08-22

**Authors:** Kevin L. Shores, George A. Truskey

**Affiliations:** Department of Biomedical Engineering, Duke University, Durham, NC, United States

**Keywords:** progeria, progerin, lamin a, mechanotransduction, endothelial cell, vascular smooth muscle cell, atherosclerosis

## Abstract

Hutchinson-Gilford Progeria Syndrome (HGPS) is a premature aging disorder that causes severe cardiovascular disease, resulting in the death of patients in their teenage years. The disease pathology is caused by the accumulation of progerin, a mutated form of the nuclear lamina protein, lamin A. Progerin binds to the inner nuclear membrane, disrupting nuclear integrity, and causes severe nuclear abnormalities and changes in gene expression. This results in increased cellular inflammation, senescence, and overall dysfunction. The molecular mechanisms by which progerin induces the disease pathology are not fully understood. Progerin’s detrimental impact on nuclear mechanics and the role of the nucleus as a mechanosensor suggests dysfunctional mechanotransduction could play a role in HGPS. This is especially relevant in cells exposed to dynamic, continuous mechanical stimuli, like those of the vasculature. The endothelial (ECs) and smooth muscle cells (SMCs) within arteries rely on physical forces produced by blood flow to maintain function and homeostasis. Certain regions within arteries produce disturbed flow, leading to an impaired transduction of mechanical signals, and a reduction in cellular function, which also occurs in HGPS. In this review, we discuss the mechanics of nuclear mechanotransduction, how this is disrupted in HGPS, and what effect this has on cell health and function. We also address healthy responses of ECs and SMCs to physiological mechanical stimuli and how these responses are impaired by progerin accumulation.

## Introduction

Mechanotransduction is the translation of mechanical stimuli into biochemical signals through numerous mechanosensitive cellular components such as stretch-activated ion channels, G protein-coupled receptors (GPCRs), integrins, and cytoskeletal filaments. The cellular response to mechanical forces is critical for tissue development and homeostasis (Humphrey et al., 2014; Davis et al., 2023). By decoupling nuclear and cytoplasmic responses to external mechanical stresses, a role for the nucleus as a mechanosensor has been identified (Kirby and Lammerding, 2018). Nuclear mechanotransduction involves the generation and regulation of signals transmitted through the cytoskeleton or modulation of gene expression by nuclear deformation. How the nucleus senses mechanical stimuli and what effect that has on cellular function is of particular interest in the context of various diseases, specifically those that affect nuclear architecture like laminopathies. These are genetic disorders that cause mutations in proteins of the nuclear lamina, leading to structural disruption of the nucleus or gene mis-regulation (Maraldi et al., 2011). Some of the most studied laminopathies include Emery-Dreifuss muscular dystrophy, dilated cardiomyopathy, and the accelerated aging disorder, Hutchinson-Gilford Progeria Syndrome (HGPS) (Maurer and Lammerding, 2019). Of these, HGPS is the most severe, resulting in the premature death of patients in their early teens due to extreme cardiovascular disease caused by progerin, a truncated and farnesylated form of the lamin A protein (Gordon et al., 2014). Cells that are exposed to high, continuous mechanical stresses are often the most affected in HGPS. These include bone (Schmidt et al., 2012), skin (Sagelius et al., 2008), and vascular cells, particularly the smooth muscle cells (SMCs) within larger arteries (Murtada et al., 2023; Olive et al., 2010). Vascular cells are constantly exposed to varying mechanical stresses from blood flow and intraluminal pressure, and thus are highly sensitive to perturbations in these forces (Davis et al., 2023). Disturbances in mechanical loads within the vasculature, which occur within curved or branched arteries, can promote cellular dysfunction, leading to manifestation of disease (Chiu and Chien, 2011). Impairment of cellular responsiveness to physiological mechanical signaling may contribute to cellular dysfunction and disease in HGPS (Hennekam, 2006). Here, we discuss the influence of progerin expression on cellular mechanotransduction to fluid and solid stresses acting on vascular cells and evaluate how this might contribute to the vascular pathology seen in HGPS.

## Pathophysiology of Hutchinson-Gilford Progeria Syndrome

HGPS is a rare and fatal disease that results in premature aging of children. Common symptoms of the disease include delayed growth, alopecia, bone dysplasia, joint contracture, scleroderma, lipodystrophy, and severe atherosclerosis (Olive et al., 2010; Gordon et al., 2007; Merideth et al., 2008; Ullrich and Gordon, 2015). The atherosclerosis, manifested as vascular stiffening, calcification, and fibrotic thickening of the vessel wall, leads to myocardial infarction or stroke. The disease develops rapidly with patients only surviving into their mid-teens (Olive et al., 2010; Hennekam, 2006; Gordon et al., 2014).

In over 90% of the affected population, the disease pathology is caused by a point mutation in the *LMNA* gene (c. 1824C>T) (Eriksson et al., 2003; De Sandre-Giovannoli et al., 2003). This mutation disrupts the post-translational processing of pre-lamin A, a major component of the nuclear lamina, by activating a cryptic splice site, causing an in-frame deletion of 50 amino acids near the C-terminus. Under healthy conditions, prelamin A is post-translationally modified by two transfer reactions, adding a farnesyl and carboxymethyl group, and two proteolytic cleavages that remove these groups to form mature lamin A (Musich and Zou, 2009). The mutated protein in HGPS, called progerin, lacks sites for endoproteolytic cleavage and remains farnesylated and carboxymethylated (Ahmed et al., 2018; Eriksson et al., 2003). At the cellular level, this leads to increased inflammation (Osorio et al., 2012), accelerated senescence (Cao et al., 2011; Wheaton et al., 2017; Benson et al., 2010), and stem cell dysfunction (Scaffidi and Misteli, 2008; Espada et al., 2008; Rosengardten et al., 2011). Progerin accumulates in the nuclear lamina causing abnormal nuclear shapes and protrusions (“blebs”) (Goldman et al., 2004), defects in DNA repair mechanisms (Gonzalo and Kreienkamp, 2015), epigenetic alterations (Shumaker et al., 2006), loss of heterochromatin (Goldman et al., 2004; McCord et al., 2013), and nuclear stiffening (Booth et al., 2015). While it is not yet fully understood how progerin causes cellular and nuclear dysfunction, a disruption to mechanotransduction appears to play a significant role.

## Nuclear mechanotransduction and the impact of laminopathies

**FIGURE 1:**
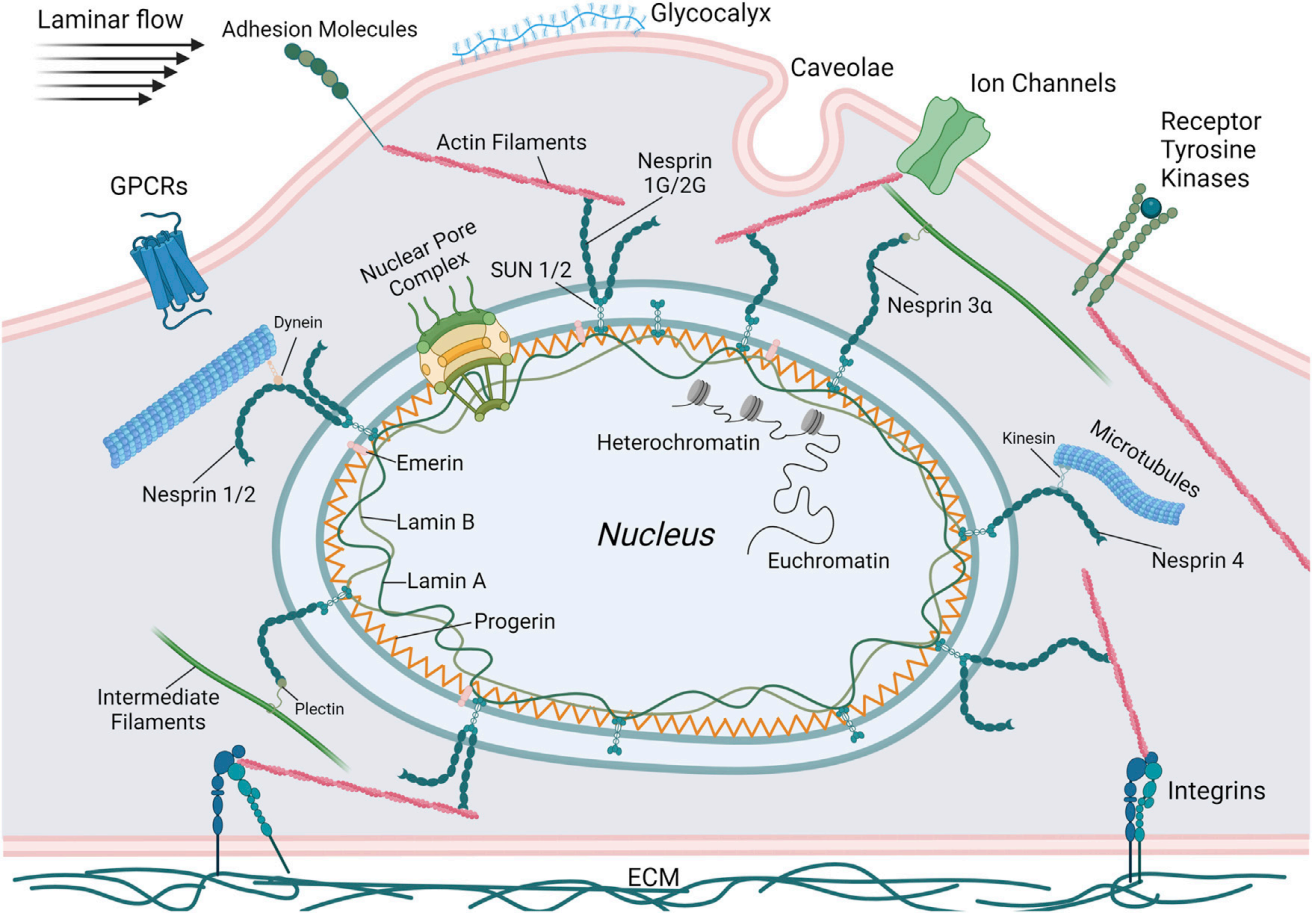
Schematic illustrating the primary mechanosensing elements of ECs including G-protein coupled receptors (GPCRs), adhesion molecules, ion channels, receptor tyrosine kinases, integrins, caveolae, and the glycocalyx. Also depicted are the components of the LINC complex. Lamins and, in the case of HGPS, progerin, are localized to the nuclear periphery and bind DNA or transcription factors. SUN proteins bridge the nuclear membrane, connecting intranuclear lamins with extranuclear KASH proteins (nesprins 1-4). These nesprins then bind to the cytoskeletal elements actin, microtubules, and intermediate filaments that are connected to or influenced by the various mechanosensors. Thus, the complete mechanotransductive pathway that regulates gene expression in response to external mechanical stresses can be visualized. This figure was generated using BioRender.

Mechanotransduction within the nucleus occurs through the nuclear envelope. This structure is comprised of the outer nuclear membrane (ONM), the inner nuclear membrane (INM), the perinuclear space between the ONM and INM, nuclear pore complexes (NPCs), and the nuclear lamina (Kalukula et al., 2022). The nuclear envelope is connected to the cytoskeleton via the linker of nucleoskeleton and cytoskeleton (LINC) complex. Actin filaments, microtubules, or intermediate filaments in the cytoplasm bind directly or indirectly to nesprin proteins that are localized to the ONM (Figure 1). Within the perinuclear space, these nesprins bind SUN (Sad1p, UNC-84) domain-containing proteins that span the INM and connect to the lamins of the nuclear lamina, NPCs, or directly to chromatin (Crisp et al., 2006; Kalukula et al., 2022). The nuclear lamina, comprised of A-type lamins (lamin A and C) and B-type lamins (lamin B1 and B2) bind with NPCs, LINC complex proteins, and chromatin (Burke and Stewart, 2013; de Leeuw et al., 2018). These elements provide a direct link from the cytoskeleton to DNA and facilitate changes in gene expression that occur in response to mechanical stimuli. The role of the LINC complex in vascular mechanotransduction was recently reviewed (Bougaran and Bautch, 2024) and only key features will be noted here.

The nuclear lamina is essential for maintaining nuclear structure and organization of the genome (Gruenbaum and Foisner, 2015). Lamins provide a structural scaffold, anchoring chromatin and transcription factors to the nuclear periphery. This anchoring facilitates the compartmentalization of the genome, which is essential for accurate DNA transcription, replication, and repair. Furthermore, lamin expression levels are directly associated with nuclear mechanical stability, as well as tissue rigidity and plasticity. Higher amounts of lamin A and C correlate with increased nuclear stiffness, while lower amounts result in more deformable and fragile nuclei (Maurer and Lammerding, 2019). Lamins recruit DNA damage response machinery and regulate transcription factor binding. They also modulate heterochromatin, domains of densely packed DNA that are transcriptionally repressed and anchor it to the nuclear periphery (Camozzi et al., 2014). Specifically, lamin A interacts with INM proteins to regulate the location of heterochromatin based on extracellular mechanical stresses (Capanni et al., 2009; Maraldi et al., 2011; Solovei et al., 2013). Lamins also bind and anchor transcription factors to the nuclear periphery, regulating expression of the genes associated with these factors (Gruenbaum and Foisner, 2015). Dysfunctional lamin A, like that seen in HGPS, can detrimentally alter its ability to maintain appropriate nuclear mechanical properties and regulate gene expression.

In addition to lamins regulating gene expression through the organization of heterochromatin and transcription factors, there are several other ways that mechanical forces influence the activation or repression of genes. Mechanical stresses can induce nuclear deformations that result in immediate stretching of chromatin through the connections of the cytoskeleton and LINC complex (Tajik et al., 2016). While not completely understood, the presiding theory is that chromatin opening reveals previously inaccessible areas of the genome, resulting in rapid transcription of mechanosensitive genes (Dupont and Wickström, 2022; Kirby and Lammerding, 2018). In addition to the direct stretching of chromatin, these nuclear deformations can also stretch the NPCs localized within the nuclear envelope. NPCs allow the exchange of macromolecules between the nucleus and cytoplasm. NPCs are governed by the binding of soluble nuclear transport factors and transport signals (e.g., nuclear localization sequences or nuclear export sequences) to macromolecules that then allow docking to and transport through the NPC (Strambio-De-Castillia et al., 2010). Stretching of the nuclear membrane increases the diameter of the NPC opening, allowing import of mechanoresponsive transcriptional regulators like YAP (Yes-associated protein) (Elosegui-Artola et al., 2017). Depending on the nature of the mechanical force and the resultant deformation of the nuclear membrane, NPC stretching can promote nuclear export, or the NPC opening can constrict, inhibiting exchange of macromolecules (Kalukula et al., 2022). The nuclear membrane also controls stretch-sensitive ion channels that can be opened during nuclear deformations induced by mechanical stresses. These channels allow increased uptake of calcium into the nucleus, altering chromatin organization (Nava et al., 2020). Finally, external forces can induce conformational changes and increase phosphorylation of lamins (Buxboim et al., 2014b; Swift et al., 2013). Lamin conformational changes can expose residues, altering interactions with other proteins and downstream signaling (Ihalainen et al., 2015), while lamin phosphorylation influences their nucleo-cytoskeletal coupling (Guilluy et al., 2014), chromatin binding (Liu and Ikegami, 2020), and regulation of nuclear stiffness through lamin disassembly (Buxboim et al., 2014a). Due to truncation of amino acids, progerin lacks some of the major phosphorylation sites found on lamin A (Ao et al., 2023). This reduces its rate of phosphorylation in response to mechanical stimuli (Cho et al., 2018) and promotes cellular senescence (Ao et al., 2023).

Laminopathies like HGPS result from mutations that alter lamin function or expression. There are two prevailing hypotheses as to how these dysfunctional lamins cause disease: (1) mutant lamins make the nucleus more fragile, resulting in greater nuclear ruptures and cell death, particularly in tissues exposed to high mechanical stresses, and (2) lamin mutations alter gene expression through impaired chromatin interactions or inhibition of transcription factor binding (Maurer and Lammerding, 2019). Nuclear mechanotransduction can influence both proposed mechanisms. Cells with lamin A mutations or deletions exhibit increases in nuclear ruptures following mechanical loading that leads to greater cell death (Lammerding et al., 2004; Kim et al., 2021; Earle et al., 2020; Kim et al., 2024; Sears and Roux, 2022). Of note, decreased levels of lamin A correspond to more deformable nuclei with increased fragility, making them more susceptible to rupture (Lammerding et al., 2004). In contrast, some lamin A mutations, like those that produce progerin, increase nuclear stiffness (Dahl et al., 2006), reducing its compliance and ability to deform under mechanical loading, resulting in a greater susceptibility to rupture (Kim et al., 2021; Yue et al., 2023; Verstraeten et al., 2008). Preceding cell death, nuclear rupture can also promote senescence and the production of pro-inflammatory cytokines known as the senescence-associated secretory phenotype (SASP). Genomic DNA released into the cytosol following nuclear rupture binds to cyclic GMP-AMP synthase (cGAS), which triggers SASP production (Dou et al., 2017; Glück et al., 2017). HGPS and other laminopathies also cause DNA damage. This can occur through dysfunctional nuclear-cytoskeletal coupling, in the form of nuclear membrane rupture (Kalukula et al., 2022), that causes impaired recruitment (Liu et al., 2005) or cytoplasmic mislocalization (Xia et al., 2018) of DNA damage response factors.

Progerin can change the epigenetic landscape by altering the state of histone methylation, specifically reducing methylation of histone 3 on lysine 9 (H3K9) and H3K27, while increasing methylation of H4K20 (Shumaker et al., 2006; Columbaro et al., 2005). Progerin is also involved in the regulation of histone deacetylases (HDACs), with HGPS cells showing a loss of HDAC1 that correlated with heterochromatin defects (Pegoraro et al., 2009). While these epigenetic changes seen with HGPS have yet to be directly correlated with aberrant nuclear mechanotransduction, there is evidence for their regulation in response to mechanical stimuli under other conditions (Kalukula et al., 2022). Changes to nuclear mechanics induced by progerin accumulation may contribute to the apparent epigenetic alterations. H3K9me3 is suppressed in cardiomyocytes and dissociates from the nuclear periphery during environmental stiffening that reduces nuclear deformations, presumably increasing nuclear stiffness (Seelbinder et al., 2021). A similar mechanism of epigenetic alteration could be imagined in HGPS, where progerin accumulation leads to nuclear stiffening and an impairment of its response to mechanical stimuli (Booth et al., 2015), leading to a reduction in histone methylation. These detriments to nuclear structure and genome stability seen in HGPS are especially prevalent in cells under constant biomechanical stress, like those of the vasculature. Moreover, progerin accumulation within the nucleus can alter mechanotransduction pathways leading to cellular dysfunction.

## Mechanotransduction in healthy endothelial cells

The arterial endothelium is exposed to two primary stresses (force per unit area) in the body: shear stress from the flow of blood and circumferential stress due to stretching of arteries from increases in blood pressure (Hahn and Schwartz, 2009). Responses to these forces modulate critical homeostatic functions such as inflammation, vasomotor tone, and vascular remodeling (Chiu and Chien, 2011). ECs sense these forces in a variety of ways such as ion channels (e.g., Piezo1, TRPV4, inner-rectifier K^+^ channel), G protein-coupled receptors (GPCRs), tyrosine kinase receptors (e.g., VEGFR2), caveolae, the glycocalyx, and integrins (Figure 1). (See (Davis et al., 2023) for an extensive review). Increases in laminar shear stress, brought on by increasing blood flow due to altered physical activity such as exercise, induce ECs to generate prostacyclin and nitric oxide (NO). These molecules promote relaxation of smooth muscle cells (SMCs) in the vascular media, providing control over vasomotor tone and ultimately blood pressure (Davies, 1995). Prostacyclin, additionally, promotes SMC health and function by increasing transcription of transgelin (SM22α), which results in a more contractile SMC phenotype (Tsai et al., 2009). NO is generated in response to shear stress through the platelet/endothelial cell adhesion molecule 1 (PECAM1)/vascular endothelium (VE)-cadherin/vascular endothelial growth factor receptor 2 (VEGFR2) complex. Initially, PECAM1 is phosphorylated in response to flow and activates a Src family kinase. VE-cadherin associates with VEGFR2 (also known as FLK1) and brings it into proximity with PECAM1. VEGFR2 is then activated by the Src family kinase and proceeds to recruit and activate phosphoinositide 3-kinase (PI3K). PI3K then phosphorylates endothelial nitric oxide synthase (eNOS) in an Akt-dependent manner. This phosphorylated eNOS, through a series of redox reactions, produces NO to relax SMCs and dilate the vessel (Tzima et al., 2005). ECs can also modulate vascular tone by releasing potassium through calcium-sensitive potassium channels that transport the potassium to the SMCs, triggering hyperpolarization and relaxation (Garland and Dora, 2017).

In addition to generating NO, shear forces also cause ECs to align in the direction of flow. This reorganization of the cytoskeleton is mediated by the PECAM1/VE-cadherin/VEGFR2 complex as well. Specifically, PI3K promotes conformational activation of integrins that, in turn, activate small GTPases (Rac, Rho, and CDC42), which regulate cell-cell junctions and remodeling of the actin cytoskeleton to elongate and align ECs in the flow direction (Osborn et al., 2006; Hahn and Schwartz, 2009). The cytoskeleton is critical for EC sensing and reacting to external stimuli as inhibiting actin, microtubules, or intermediate filaments block many EC responses to flow (Hahn and Schwartz, 2009).

Shear stresses have a direct effect on the nucleus and LINC complex in ECs. Cytoskeletal reorganization under flow leads to protrusion of the nucleus, exposing it to higher shear stresses than the rest of the cell (Tkachenko et al., 2013; Barbee et al., 1994; Barbee et al., 1995). Like the cell body cytoskeleton, the nucleus elongates in the direction of flow, providing a visual confirmation for its mechanosensing capacity (Danielsson et al., 2022b; Nava et al., 2020; Deguchi et al., 2005). Within the nuclear envelope, the LINC complex proteins exhibit essential functions in regulating many EC mechanotransductive pathways. SUN1 and 2 regulate nesprin-1 interactions with microtubules, which influences signaling of the small GTPase Ras homology family (Rho) proteins that alter cell-cell junctions or induce expression of EC tight junction proteins, leading to increased barrier function (Yang et al., 2020; Buglak et al., 2023).

In ECs, many transcription factors and transcriptional coactivators are upregulated in response to laminar shear stresses. These, along with the alteration of histone acetylation and phosphorylation induced by flow (Chen et al., 2008; Wang et al., 2010; Chen et al., 2010), modulate gene expression. Krüppel-like Factor (KLF) 2 and 4 and nuclear factor erythroid 2-related factor 2 (NRF2) are transcription factors critical to maintaining EC homeostasis. KLF2 expression is upregulated by phosphorylation and nuclear export of histone deacetylase 5 (HDAC5) in response to shear. Specifically, KLF2 induces eNOS expression and inhibits pro-inflammatory signaling through suppression of vascular cell adhesion molecule 1 (VCAM1) and E-selectin. KLF2 recruits the histone acetyltransferase p300 and outcompetes nuclear factor-κB (NF-κB), which promotes the expression of inflammatory genes (SenBanerjee et al., 2004; Dekker et al., 2002). In addition to increased recruitment, hemodynamic shear stress increases the activity of the transcriptional coactivator p300, which leads to increased eNOS transcription (Chen et al., 2008). YAP is another transcriptional coactivator that regulates many functions in ECs. Its phosphorylation leads to its proteasomal degradation in the cytosol, while dephosphorylation results in nuclear localization and activation of genes (Dupont et al., 2011). YAP nuclear localization is increased by the resultant NPC opening from mechanically stretching cells (Elosegui-Artola et al., 2017). Its translocation is also dependent on nuclear compression. Nuclei that became softer after silencing of lamin A/C experienced greater compression and elevated YAP nuclear localization (Koushki et al., 2023). Laminar flow promotes YAP phosphorylation in ECs, inhibiting its nuclear localization, through activation of G-protein-integrin interactions and RhoA inhibition (Wang et al., 2016a). YAP suppression downregulates pro-inflammatory gene expression such as monocyte chemoattractant protein-1 (MCP1), intercellular adhesion molecule-1 (ICAM1), and vascular cell adhesion molecule-1 (VCAM1) (Liu et al., 2019; Xu et al., 2016; Wang et al., 2016b). In contrast, YAP activation promotes inflammation and proliferation in ECs (Wang et al., 2016a). How the shear stress response of ECs is disrupted in HGPS is not completely understood, but several studies have started to reveal possible mechanisms that relate EC dysfunction with an impaired mechanosensing ability.

## Disruption of mechanotransduction in HGPS endothelial cells

Many of the homeostatic functions of ECs discussed in the previous section are diminished or even reversed under disturbed flow that generates low, oscillatory shear stresses around vessel branches or in curved arteries (Andueza et al., 2020; Jiang et al., 2014; Lee et al., 2012) (Table 1). When exposed to disturbed flow, ECs lose their ability to regulate vasomotor tone by inducing SMC relaxation and vessel dilation (Chiu and Chien, 2011). This is caused by a reduction in KLF2/4, which activates eNOS and NO production under physiological laminar shear (Andueza et al., 2020; Jiang et al., 2014). Phosphorylation of eNOS is also reduced due to inhibition of the mechanosensitive adenosine monophosphate-activated protein kinase (AMPK) pathway (Guo et al., 2007). Disturbed flow increases EC inflammation due to reduced KLF2 expression (SenBanerjee et al., 2004), increased YAP/TAZ nuclear localization (Wang et al., 2016a; Wang et al., 2016b), and enhanced NF-κB activity (Nagel et al., 1999). It also reduces autophagic flux (Vion et al., 2017) and increases endoplasmic reticulum stress (Civelek et al., 2009). Additionally, ECs lose their migratory capacity and exhibit impaired alignment with the flow direction due to, in part, a disruption of nuclear-cytoskeletal coupling by loss of nesprin-1 (Chancellor et al., 2010; Denis et al., 2021; King et al., 2014). Moreover, low shear stresses (5 dynes/cm^2^) decrease nesprin −2 and lamin A in ECs, which leads to an increase in apoptosis (Han et al., 2015).

**TABLE 1:** Summary of the major findings from studies showing a disrupted mechanotransduction pathway in ECs expressing progerin.

Model	Major findings	Reference
*in vitro* (HUVECs expressing progerin)	• Significant EC loss and increase in apoptosis markers under physiological shear stress • EC death was rescued by FTI treatment or pre-aligning HUVECs to flow direction	Danielsson et al. (2022a)
*in vitro* (HUVECs expressing progerin)	• Physiological shear stress had no effect on chromatin dynamics, possibly due to increased nuclear stiffness	Booth et al. (2015)
*in vitro* (iPSC-derived ECs from HGPS patients)	• Lower eNOS expression under physiological flow • Increased E-selectin and VCAM1 expression	Atchison et al. (2020)
*in vivo* (EC-specific progerin expression mouse model)	• Elevated levels of ICAM1 in lung endothelium • Reduced eNOS levels and NO production • Significantly reduced Sirt7 levels • Treatment with AAV encoding Sirt7 increased average lifespan by 76%	Sun et al. (2020)
*in vivo* (EC-specific progerin expression mouse model)	• ECs in descending aorta did not align with blood flow • Reduced levels of eNOS, likely from accumulation of MRTFA at nuclear periphery, and NO production • Treatment with MRTFA inhibitor rescued eNOS levels	Osmanagic-Myers et al. (2019)

HUVECs, human umbilical vein endothelial cells; FTI, farnesyltransferase inhibitor; iPSC, induced pluripotent stem cell; VCAM1, vascular cell adhesion molecule 1; ICAM1, intercellular adhesion molecule 1; eNOS, endothelial nitric oxide synthase; NO, nitric oxide; AAV, adeno-associated virus; MRTFA, myocardin-related transcription factor A.

When exposed to the more uniform laminar flow conditions in straight regions of blood vessels, HGPS ECs mirror the dysfunction seen under disturbed flow, indicating an inability to respond to external mechanical stimuli, presumably because of progerin accumulation within the nucleus. Several *in vitro* studies have shown HGPS EC dysfunction in response to laminar shear stress. Danielsson and colleagues expressed progerin in human umbilical vein endothelial cells (HUVECs) and exposed them to physiological shear (12 dynes/cm^2^) for 3 days. They found significant cell loss and increased markers of apoptosis compared to control HUVECs (Danielsson et al., 2022a). These responses were rescued by treatment with a farnesyltransferase inhibitor (FTI), that prevents progerin accumulation at the nuclear periphery (Yang et al., 2006), or, interestingly, by pre-aligning the HUVECs to flow prior to inducing progerin expression (Danielsson et al., 2022a). This suggests that progerin accumulation inhibits ECs ability to respond to flow, and that this impairment can result in increased EC apoptosis. HGPS ECs have lower eNOS expression after exposure to steady laminar shear stress for 24 h (Atchison et al., 2020; Gete et al., 2021), increased inflammation (Atchison et al., 2020; Osmanagic-Myers et al., 2019; Sun et al., 2020), and an inability to properly align under physiological flow (Osmanagic-Myers et al., 2019; Danielsson et al., 2022a). Treatment of HGPS ECs with the FTI lonafarnib or the rapamycin analog Everolimus restored eNOS and KLF2 expression (Abutaleb et al., 2023).

Another study measured chromatin dynamics under 20 dynes/cm^2^ shear stress in HUVECs treated with exogenously expressed progerin (Booth et al., 2015). External forces had no effect on chromatin dynamics in HUVECS expressing progerin compared to untreated HUVECs, possibly due to the increased nuclear stiffness caused by progerin accumulation (Verstraeten et al., 2008). This suggests a disruption in the nuclear mechanotransduction pathway that can alter chromatin organization, and possibly inhibit the regulation of critical mechanoresponsive genes.

Tissue-engineered blood vessels (TEBVs) prepared using induced pluripotent stem cell (iPSC)-derived ECs and SMCs from HGPS patients (viECs) (Atchison et al., 2020) showed suppressed dilation in response to acetylcholine (Ach) and increases in E-selectin and VCAM1 expression (Atchison et al., 2020). These results suggest a disruption in the mechanosensing pathway, inhibiting eNOS upregulation and promoting a pro-inflammatory response under laminar flow conditions.

Two *in vivo* studies using HGPS mouse models corroborated some of the findings from *in vitro* experiments. In both models, mice solely express progerin in ECs, allowing the investigation of their effects on HGPS vascular pathology without the influence from other vascular cells. Using single-cell transcriptomics analysis, Sun and colleagues found significant increases in the inflammatory response in the lung endothelium of EC-specific progerin expressing mice, with elevated levels of ICAM1 (Sun et al., 2020). They supported these findings by overexpressing progerin in HUVECs and showing significantly elevated levels of inflammatory genes. Relative to controls, progerin-expressing ECs exhibited significantly attenuated relaxation of the thoracic aorta in response to Ach and reduced eNOS levels. Sodium nitroprusside treatment, which induces SMC relaxation in an EC-independent manner (Bonaventura et al., 2008), was unaffected, suggesting the dysfunction is due to progerin-expressing ECs. In progerin-expressing ECs, levels of Sirtuin 7 (Sirt7), a nicotinamide adenine dinucleotide (NAD^+^)-dependent deacylase were reduced significantly. Further, enhanced interactions of Sirt7 and progerin compared to lamin A resulted in increased polyubiquitination of the Sirt7. Treatment with an adeno-associated viral vector encoding Sirt7 resulted in a 76% increase in average lifespan (Sun et al., 2020). While EC response to mechanical stimuli was not directly measured in this study, the detrimental effects of progerin expression, such as increased inflammation and reduced eNOS and NO production, suggest dysfunctional mechanosensing.

In another EC-specific progerin expressing mouse model (Osmanagic-Myers et al., 2019), ECs within the descending aorta were not aligned with the direction of blood flow. Progerin-expressing ECs exposed to 12 dynes/cm^2^ shear stress *in vitro* did not align with flow or exhibit nuclear elongation. Progerin-expressing ECs also showed reduced levels of eNOS, which resulted in lower systemic NO levels. To determine possible mechanisms for this dysfunctional response to shear stresses, the authors found upregulation of both SUN1 and 2 as well as an increase in actin polymerization, which may have been caused by a mislocalization of emerin (a nuclear envelope protein that regulates expression of mechanosensitive genes) into discrete aggregates (Osmanagic-Myers et al., 2019).

SUN1 expression also increased in another HGPS mouse model and human HGPS fibroblasts (Chen et al., 2012). This led to increases in nuclear defects and cellular senescence, with the reduction in heterochromatin due to increased SUN1 expression appearing to play a role. SUN1 knockdown significantly decreased cellular senescence in HGPS fibroblasts and more than doubled the lifespan of progeroid mice (Chen et al., 2012). The upregulation of SUN1 in progerin-expressing ECs, seen by Osmanagic-Myers and colleagues (Osmanagic-Myers et al., 2019), may have contributed to the dysfunctional cell response to mechanical stimuli. Additionally, a recent study evaluating the effects of cyclic stretch on mesenchymal stromal cells from a *Zmpste24* ^−/−^ mouse model supports the contribution of increased SUN2 expression and actin polymerization in promoting cellular dysfunction (Yue et al., 2023). The *Zmpste24* ^*−/−*^ model lacks the metalloprotease needed to cleave the farnesyl group from prelamin A during posttranslational processing. This results in a permanently farnesylated prelamin A that accumulates at the nuclear envelope, similarly to progerin, leading to misshapen nuclei, increased nuclear stiffness, and elevated cellular senescence (Mu et al., 2020). This model exhibited increases in SUN2 expression, actin polymerization, and nuclear stiffness that correlated with increased DNA damage and cellular senescence. SUN2 suppression reduced DNA damage and senescence following mechanical loading. This response was potentially mediated by nuclear decoupling and softening as polymerized actin levels and nuclear stiffness also decreased with SUN2 knockdown (Yue et al., 2023). Though not directly measured, an increased nuclear stiffness due to elevated SUN2 expression and subsequent actin polymerization may have influenced the impaired shear response of ECs (Booth et al., 2015; Danielsson et al., 2022a) seen by Osmanagic-Myers and colleagues (Osmanagic-Myers et al., 2019). These authors also found increased accumulation of myocardin-related transcription factor A (MRTFA) at the nuclear periphery, associated with progerin. MRTFA binds to the eNOS promoter region and reduces its expression (Fang et al., 2011). The enhanced localization of MRTFA at the nuclear periphery in progerin-expressing ECs appeared to increase its association with eNOS leading to eNOS suppression. Treating ECs with a small molecule MRTFA inhibitor (CCG-203971) rescued eNOS levels. Importantly, MRTFA is a mechanosensitive transcription factor whose nuclear localization is increased by emerin expression and subsequent actin polymerization (Osmanagic-Myers et al., 2019). Collectively, this study provides evidence for the impact of progerin expression on the ability of ECs to properly respond to mechanical stimuli. Understanding what other mechanotransduction pathways might be affected in ECs will be an important next step for determining how to mitigate their dysfunctional response.

## Mechanotransduction in healthy vascular smooth muscle cells

Vascular SMCs are exposed to intraluminal stresses caused by changes in blood pressure that lead to stretch that depends on the extent of vessel dilation and cell orientation (Davis et al., 2023). A phenomenon known as the myogenic response causes SMCs in arterioles to constrict in response to increases in luminal pressure. This response does not require an intact endothelium or innervation, although it can be influenced by them (Davis and Hill, 1999). The myogenic response has several physiological functions, primarily establishing a basal vascular tone and responding to changes in blood flow. Ion channels are most likely involved in the force sensing mechanism (see Ref (Davis et al., 2023) for an extensive review). Ion channels are activated by mechanosensing GPCRs such as angiotensin II type 1 receptor (AT1R). Cell stretching changes GPCR conformation, allowing it to activate an associated G protein (Zou et al., 2004). The activated ion channels cause a depolarization of the SMC plasma membrane, which stimulates voltage-gated calcium channels, leading to an increase in intracellular calcium. The increased calcium activates myosin light chain 20 (MLC20), resulting in vasoconstriction (Davis et al., 2023). In addition to ion channels, GPCRs activate enzymes such as Ras homology family A (RhoA). This enzyme controls the activity of several downstream effector proteins that impact SMC proliferation, migration, differentiation, and contractility. For example, the effector protein, Rho associated coiled-coil containing protein kinase 2 (ROCK2) alters contractility by promoting phosphorylation of MLC20 (de Godoy and Rattan, 2011).

Mechanical forces significantly influence gene expression and phenotype changes in SMCs, particularly under pathological conditions (Owens et al., 2004). *In vitro*, physiological stretching of cells (≤10%) results in maintenance of a contractile phenotype (Mao et al., 2012; Reusch et al., 1996; Yao et al., 2014; Huang et al., 2015), while supraphysiological stretching decreases the expression of contractile genes encoding proteins such as calponin, transgelin, α-smooth muscle actin, and myosin heavy chain 11 (Wang et al., 2018; Wan et al., 2015; Hu et al., 2014). Upregulation of contractile proteins under physiological loading appears to be matrix-dependent, with SMCs cultured on basement membrane laminin showing the greatest upregulation and those cultured on fibronectin showing no upregulation (Reusch et al., 1996). Correspondingly, cyclic stretching regulates integrin expression to influence interactions with extracellular matrix (ECM) and downstream mechanotransduction pathways (Mao et al., 2012).

Transforming growth factor β and sirtuin pathways are implicated in the regulation and maintenance of a contractile SMC phenotype (Yao et al., 2014; Huang et al., 2015). HDACs also contribute to SMC phenotype regulation, with HDAC7 being upregulated and HDACs 3 and 4 being downregulated in a more contractile, quiescent phenotype (Yan et al., 2009). Changes to the SMC phenotype were influenced by both matrix stiffness and stretching through regulation of RhoA-ROCK2 and calcium influx pathways, respectively (Swiatlowska et al., 2022). Discoidin domain receptor-1 (DDR1) is a membrane protein that acts as a mechanosensor after binding to collagen (Ngai et al., 2020). Increased stiffness of the ECM, which occurs as HGPS progresses, leads to increased expression and activation of DDR1. This leads to YAP activation (Ngai et al., 2022) and stress fiber formation and expression of genes involved in calcification. Further, YAP activation leads to further DDR1 expression creating a positive feedback loop promoting vessel stiffness (Ngai et al., 2022).

## Disruption of mechanotransduction in HGPS smooth muscle cells

While HGPS ECs clearly show some level of dysfunction in response to physiological shear stresses, the substantial decrease in the number of SMCs within the medial layer of large arteries (Varga et al., 2006) is believed to be the driver of the cardiovascular dysfunction in HGPS. As such, many studies have attempted to determine the mechanism behind this significant reduction in cellular content unique to the vascular media. Although the loss of SMCs is the predominant feature associated with HGPS pathology, there is also an SMC dysfunction that appears to be attributed, at least in part, to altered mechanotransduction, including increased inflammation (Ribas et al., 2017), elevated DNA damage (Kim et al., 2021), and reduced contractile response (de Godoy and Rattan, 2011; Murtada et al., 2020) (Table 2). While HGPS SMCs do manifest a dysfunctional contractile response (Del Campo et al., 2020; Murtada et al., 2020), the myogenic response and SMC phenotype switching in HGPS have not been examined.

**TABLE 2:** Summary of the major findings from studies showing a disrupted mechanotransduction pathway in SMCs expressing progerin.

Model	Major findings	Reference
*in vitro* (iPSC-derived SMCs from HGPS patients)	• Increased IL-1β and IL-6 expression and elevated levels of DNA damage after 16% cyclic stretch for 24 h • Treatment with FTI or statin reduced inflammatory response under cyclic stretching	Ribas et al. (2017)
*in vivo* (G608G BAC mouse model of HGPS that expresses human progerin)	• At 12 months, significant SMC loss in ascending aorta but not descending aorta • Reduction of intermediate filament protein vimentin in SMCs of ascending aorta	Song et al. (2014)
*in vivo* (G609G mouse model of HGPS that expresses mouse progerin)	• Fewer SMCs at regions of disturbed flow within the aorta • Disrupting the LINC complex increased SMC content in the ascending aorta by 65%	Kim et al. (2018)
*in vitro* (mouse aortic SMCs expressing human progerin)	• Increasing lamin B1 led to 80% reduction in nuclear membrane ruptures, lower DNA damage levels, and increased cell survival after stretching • Elevated lamin B1 decreases nuclear stiffness by 23%	Kim et al. (2021)

FTI, farnesyltransferase inhibitor; iPSC, induced pluripotent stem cell.

Exposing SMCs derived from iPSCs of HGPS donors to 16% cyclic stretch for 24 h resulted in an increased inflammatory response, through upregulation of IL-1β and IL-6, as well as elevated levels of DNA damage (Ribas et al., 2017). While 16% stretch is generally considered pathological (Jensen et al., 2021), these deleterious results were not seen in non-HGPS controls, indicating the HGPS SMCs have a comparatively dysfunctional mechanoresponse to cyclic stretching. Treatment of these SMCs with an FTI or statin attenuated the inflammatory response to mechanical forces, suggesting progerin expression plays a role in this response. While no direct mechanism was determined for the increased inflammation, HGPS cells cultured under static conditions did not express the inflammatory markers (Ribas et al., 2017). Several *in vivo* studies using HGPS mouse models have also shown increased SMC dysfunction, though not always in the context of impaired mechanotransduction. One study that focused on the biomechanical changes of the vasculature with disease progression found, in addition to significant SMC loss, there was a total loss of vessel constriction capacity (Murtada et al., 2020) in response to phenylephrine (PE), as opposed to increased luminal pressure. However, PE mediates contraction through the RhoA-ROCK pathway, which is reduced in HGPS models (Hale et al., 2008), and, as noted, RhoA-ROCK suppression induces a more synthetic phenotype in SMCs (de Godoy and Rattan, 2011). In addition, mice in which only SMCs express progerin, exhibited significantly lower vessel constriction by PE or KCl compared with control mice that did not express progerin (Del Campo et al., 2020). Such a response was not observed in mice that expressed progerin in ECs alone.

**FIGURE 2:**
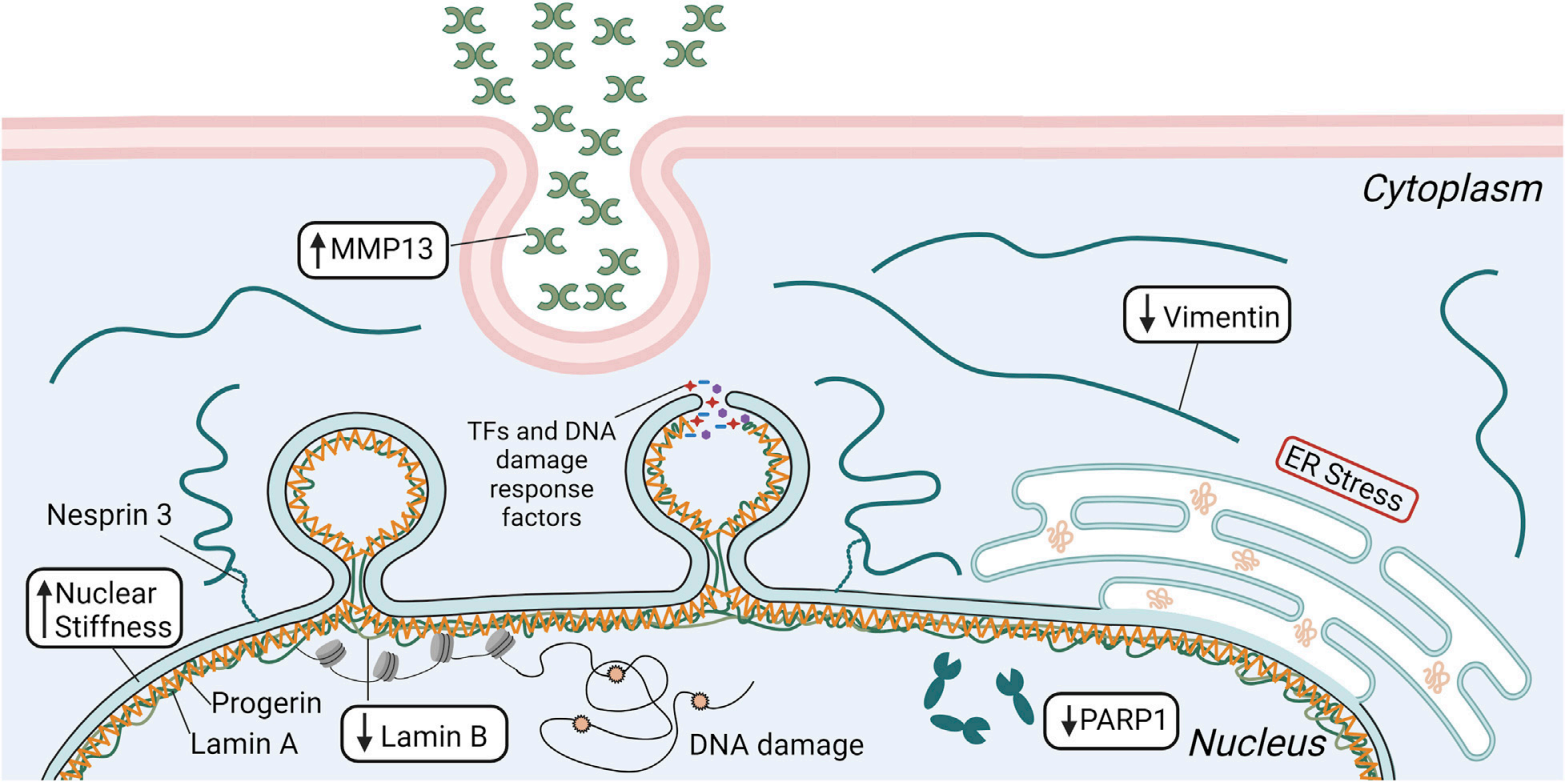
Schematic depicting several mechanisms that are involved in the significant SMC loss seen in HGPS, including elevated matrix metalloproteinase 13 (MMP13) secretion and endoplasmic reticulum (ER) stress, reduced vimentin and poly [ADP-ribose] polymerase 1 (PARP1) expression, and decreased levels of lamin B. Lower lamin B in concert with progerin accumulation increases nuclear stiffness, leading to greater incidence of nuclear rupture and DNA damage that ultimately results in increased SMC apoptosis. This figure was generated using BioRender.

*In vivo* studies observed significant SMC loss and vascular remodeling in the ascending aorta of a mouse model expressing human progerin after 12 months (Song et al., 2014). Interestingly, SMC loss did not occur in the descending aorta. The authors hypothesized this was due to the increased mechanical stresses within the ascending aorta, determining that the reduction in lumen diameter due to vascular remodeling of the ascending aorta could lead to significantly higher mechanical stresses not experienced by the descending aorta or within the ascending aorta of healthy controls (Song et al., 2014). Others have also seen differences in disease severity between different vascular regions, with the areas exposed to the highest mechanical forces being most affected (Murtada et al., 2023). In addition to significant SMC loss, there was also a reduction in intermediate filament proteins, particularly vimentin (Song et al., 2014) (Figure 2). These are important to many mechanotransduction pathways and help disperse mechanical stresses in cells (Hu et al., 2019). The descending aorta did not show the same vimentin reduction. In an *ex vivo* experiment using a microfluidic system, the descending aortas of WT and HGPS mice were exposed to high levels of shear stress (75 dynes/cm^2^). The WT samples did not show any changes in vimentin expression, but HGPS samples had a substantial reduction (Song et al., 2014). This study suggests that SMC loss seen in HGPS is dependent on the magnitude of pathological mechanical forces and that a potential mechanism involves the reduction in mechanotransduction related proteins.

Another *in vivo* study found a connection among components of the nucleoskeleton, pathological biomechanical forces, and loss of SMCs in an HGPS mouse model (Kim et al., 2018). The HGPS phenotype (e.g., greater ECM deposition and adventitial thickening with fewer SMCs) was more severe at regions of disturbed flow within the aorta. To determine if HGPS led to increased sensitivity to irregular mechanical stresses, progerin-expressing SMCs were stretched *in vitro* for 24 h. Stretching caused 40% of the SMCs to detach from the flexible membrane and these were determined non-viable, whereas WT SMCs were unaffected by the same mechanical regimen. The authors hypothesized that this response to mechanical forces may be due to the low levels of lamin B1 in SMCs combined with progerin expression, while WT SMCs are protected by their high levels of lamin A. They disrupted the connection between the nuclear and cytoskeleton by overexpressing the KASH domain of nesprin-2 (a component of the LINC complex). Since nesprins interact with SUN proteins through KASH domains, overexpressing the KASH domain of nesrpin2 occupies SUN protein binding sites, preventing the connection between the nuclear and cytoskeleton and reducing the transmission of external forces to the nucleus (Kim et al., 2018). This disruption of the LINC complex greatly improved progerin-expressing SMC survival after stretching. *In vivo*, KASH overexpression increased SMC content to 65% of WT mice in the outer curvature of the ascending aorta, and, interestingly, adventitial fibrosis was eliminated. Improvements were also seen in the inner curvature of the ascending aorta, albeit, not as pronounced, where the pathology is more severe (Kim et al., 2018). This study suggests that the resilience of the nucleus to external mechanical forces is impaired by progerin expression, leading to nuclear damage and cell death. While the exact mechanism is unknown, the authors speculate it is due to the increased nuclear rigidity imparted by progerin accumulation in conjunction with the low levels of lamin B1 seen in SMCs (Kim et al., 2018).

Increasing lamin B1 in progerin-expressing SMCs leads to an 80% reduction in nuclear membrane ruptures, lower amounts of DNA damage, and increased cell survival after 2 hours of stretching. Elevated lamin B1 decreases nuclear stiffness by 23% (Kim et al., 2021). This suggests that in HGPS SMCs, lower nuclear compliance due to reduced lamin B1 and elevated progerin leads to greater incidence of nuclear membrane ruptures and cell death. *In vivo*, progerin expression increases with age, while lamin B1 decreases, and this correlates with increased nuclear membrane ruptures in the aortic SMCs of older HGPS mice (Kim et al., 2021). In healthy SMCs, lamin A forms an organized network on the nuclear membrane with openings of mean size around 0.085 µm^2^ (Kim et al., 2024). However, when the SMCs express progerin, the network is less organized with larger openings through which blebs formed (Figure 2). The blebs were associated with low levels of lamin B1. Overexpressing lamin B1 normalized the meshwork and reduced bleb formation. Collectively, these *in vivo* studies suggest that progerin accumulation leads to a dysfunction in the components of the nucleoskeleton of SMCs that impairs their ability to resist damage caused by elevated or disturbed external mechanical forces, leading to nuclear/DNA damage and cell death.

## Future directions and conclusion

It is clear from *in vitro* and *in vivo* studies that the progerin accumulation negatively impacts mechanotransduction pathways in both ECs and SMCs. To what extent this impaired mechanoresponse influences the cellular dysfunction seen in HGPS needs additional study.

For ECs, it would be interesting to investigate the effect of modulating LINC complex proteins on their response to physiological flow, such as cell alignment, inflammation, and elevated expression of homeostatic proteins like eNOS, KLF2, and NRF2. Additionally, other mechanotransduction pathways important to EC homeostasis are disrupted in non-endothelial HGPS cells (Son et al., 2024; Kubben et al., 2016). As mentioned, in ECs, the mechanosensitive AMPK pathway activates eNOS to produce NO (Guo et al., 2007). This pathway was inhibited in HGPS fibroblasts and activating it rescued the HGPS phenotype, reducing progerin expression and DNA damage, while restoring heterochromatin marks (Son et al., 2024). Similarly, NRF2, which provides antioxidant protection and suppresses inflammation in ECs (Chen et al., 2006), is mislocalized in HGPS fibroblasts to the nuclear periphery by progerin, preventing its activation. Providing activated NRF2 prevented nuclear defects, lowered progerin expression, and reduced oxidative stress (Kubben et al., 2016). These pathways should be investigated in the context of EC mechanotransduction as they may also be inhibited by progerin expression. This could provide additional explanation for the impaired mechanoresponse of HGPS ECs. Also, understanding how nuclear mechanics change in ECs with progerin accumulation and how this might differ from SMCs could provide insight into ECs ability to survive the high shear stresses in larger arteries while SMCs cannot.

While it is unclear what causes the characteristic loss of SMCs in HGPS, progerin accumulation may decrease nuclear compliance leading to increased nuclear ruptures and cell death. Song and colleagues attributed this to the loss of vimentin in SMCs of the ascending aorta that are exposed to high shear stresses (Song et al., 2014). In support of this hypothesis, others have found vimentin to be necessary for nuclear resistance to deformation under mechanical strains (Neelam et al., 2015; Patteson et al., 2019). The nuclear elasticity imparted by vimentin reduces the incidence of nuclear rupture and DNA damage (Patteson et al., 2019). Studies with cultured cells indicate that loss of vimentin is compensated by increased ECM synthesis and stiffening of the ECM (Grolleman et al., 2023), which could then explain changes in the vessel wall composition and stiffness in HGPS. Moreover, increased endoplasmic reticulum stress (Hamczyk et al., 2019), elevated matrix metalloprotease 13 expression (Pitrez et al., 2020), and increased poly (ADP-ribose) polymerase 1 expression (Zhang et al., 2014) influence SMC loss and treatment of the specific condition resulted in improved SMC survival.

Vascular dysfunction in HGPS is likely caused by multiple factors, with impaired mechanotransduction having an influence. Progerin accumulation not only affects the mechanosensing of the cell through disruption of the nuclear-cytoskeletal coupling, but can sequester transcription factors at the nuclear periphery, affecting their activity, and alter the epigenome. The long lifetime of progerin in large arteries can amplify any of these pathways (Hasper et al., 2023). While some studies involving HGPS SMCs suggest it is mainly the change in nuclear stiffness that promotes the pathological features, ones focused on ECs show protein mislocalization (e.g., MRTFA) or destabilization (e.g., Sirt7) having a prominent influence. Understanding how these two mechanisms of progerin accumulation alter cellular function in each of these vascular cells can influence what treatment methods may be required for improving the cardiovascular disease caused by HGPS.
